# Two forms of (naphthalen-1-yl)boronic acid

**DOI:** 10.1107/S2056989016012494

**Published:** 2016-08-12

**Authors:** Kayleigh Bemisderfer, Alexander Y. Nazarenko

**Affiliations:** a Chemistry Department, SUNY Buffalo State, 1300 Elmwood Ave, Buffalo, NY 14222, USA

**Keywords:** crystal structure, aryl­boronic acid, hydrogen-bond network, polymorph

## Abstract

Two polymorphs of the title compound, C_10_H_9_BO_2_, were prepared by recystallization from different solvents at room temperature. Both forms demonstrate nearly identical mol­ecular structures with all naphthalene group atoms located in one plane and all boronic acid atoms in another: the dihedral angles between these planes are 39.88 (5) and 40.15 (5)° for the two asymmetric mol­ecules of the ortho­rhom­bic form and 40.60 (3)° for the single asymmetric mol­ecule in the monoclinic form. In each extended structure, mol­ecules form dimers, connected *via* two O—H⋯O hydrogen bonds. The dimers are connected by further O—H⋯O hydrogen bonds, forming layered networks in the (001) plane and the (100) plane in the ortho­rhom­bic and monoclinic forms, respectively. The resulting layers are practically identical in both forms. However, these layers are shifted along the [010] axis in the two forms, resulting in a slightly more effective packing for monoclinic structure (packing index = 0.692) compared to the ortho­rhom­bic form (0.688).

## Abstract 2

Two polymorphs of the title compound, C_10_H_9_BO_2_, were prepared by recystallization from different solvents at room temperature. Both forms demonstrate nearly identical mol­ecular structures with all naphthalene group atoms located in one plane and all boronic acid atoms in another. In each extended structure, mol­ecules form dimers, connected *via* two O—H⋯O hydrogen bonds. The dimers are connected by further O—H⋯O hydrogen bonds, forming layered networks. The resulting layers are practically identical in both forms but are shifted along the [010] axis in the two forms, resulting in a slightly more effective packing for the monoclinic structure compared to the ortho­rhom­bic form.

## Chemical context   

Naphthalene boronic acids (α- and β-) were first synthesized by Michaelis (1894 ▸) along with other aryl­boronic acid by reaction of di­aryl­mercury with boron trichloride with subsequent hydrolysis. A more practical procedure (König & Scharrnbeck, 1930 ▸) included the reaction of naphthyl­magnesium bromide with tri-(isobut­yl)borate. In both cases, the existence of two different forms of title compound was suggested, one forming plate-like crystals and another one forming needles.
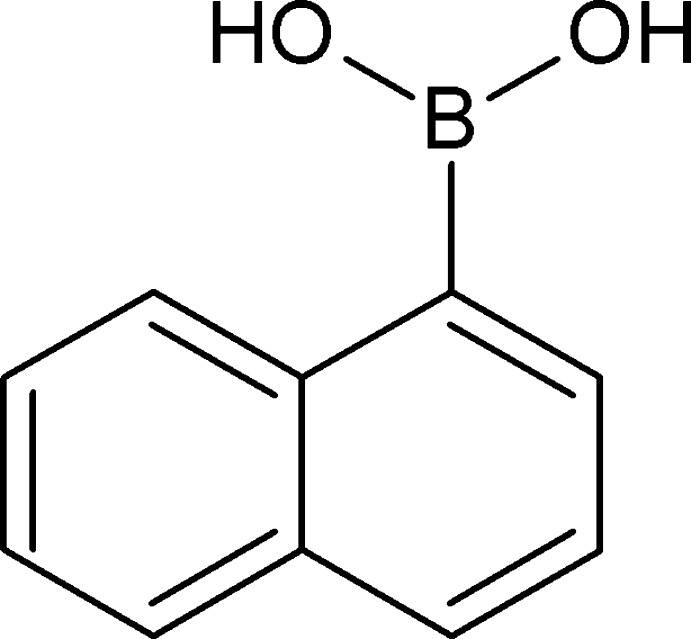


These compounds were originally investigated because of their potential in biochemistry (König & Scharrnbeck, 1930 ▸; Gao *et al.*, 2003 ▸; Hall, 2011 ▸) and later as reactants in the Suzuki reaction (Hall, 2011 ▸). 1-Naphthalene boronic acid is now commercially available and was the source for this study.

## Synthesis and crystallization   

**Figure 1:**
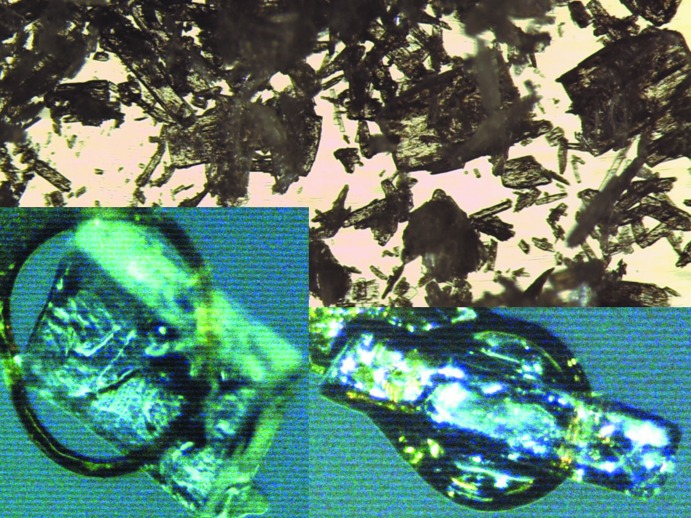
Crystals of the different polymorphs in starting material (view area 1 × 2 mm). Plate (left): ortho­rhom­bic. Needle (right): monoclinic.

A sample of 1-naphthalene boronic acid was purchased from Aldrich. Its FTIR spectrum coincided with that reported by the manufacturer. Under the microscope, a number of relatively large (up to 0.5 mm) crystals were visible, some of them suitable for single crystal X-ray data collection (Fig. 1 ▸). Experimental data revealed an ortho­rhom­bic structure for the plate-shaped crystals. Recrystallization from hot water yielded very thin plates. This polycrystalline sample showed a powder diffractogram that was slightly different from the raw material and the calculated pattern of the ortho­rhom­bic form. Attempts at slow crystallization from ethanol and toluene solution resulted in larger and better shaped crystals, some of which were ortho­rhom­bic plates and other were visibly non-ortho­rhom­bic needles (Fig. 1 ▸). Several such crystals were tested: here we report the best data for both the ortho­rhom­bic and monoclinic forms.

## Structural commentary   

**Figure 2:**
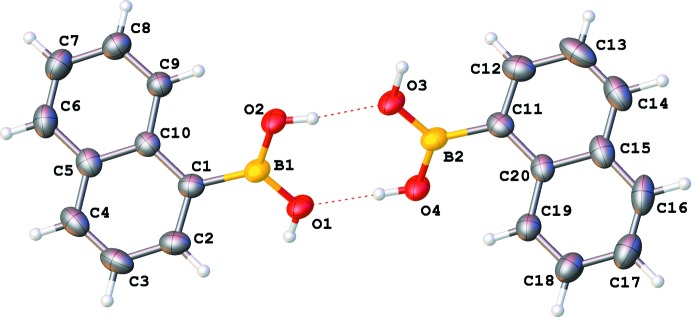
Numbering scheme of the title compound with 50% probability displacement ellipsoids (ortho­rhom­bic polymorph).

**Figure 4:**
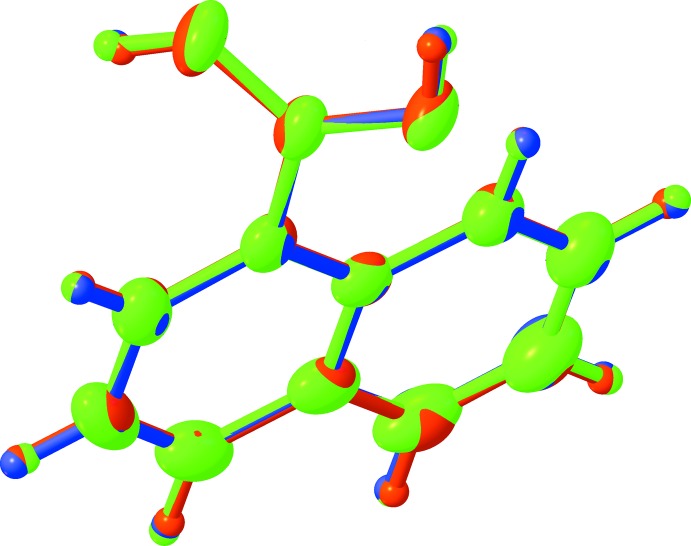
Overlay of the two polymorph mol­ecules (red & green – ortho­rhom­bic, blue – monoclinic) with appropriate inversion.

The mol­ecules of naphthalene boronic acid in both crystal structures (Figs. 2 ▸ and 3 ▸) have the usual bond distances and angles. There is one mol­ecule in the asymmetric unit of the monoclinic structure. In the non-centrosymmetric ortho­rhom­bic structure, the two mol­ecules in the asymmetric unit have very similar structures: they almost coincide (after inversion for one of them) with each other as well, as with the unique mol­ecule from the monoclinic structure (Fig. 4 ▸).

In the monoclinic structure, the mean plane of the naphthalene fragment is tilted from plane of boron and two oxygen atoms with an angle of 40.60 (3)°. The boron atom deviates by 0.0449 (16) Å from the mean plane of the naphthalene ring system.

In the ortho­rhom­bic structure, there are two independent mol­ecules. When superimposed, the angle between the mean planes of the naphthalene ring systems is only 0.88 (6)°. Two boron atoms and four oxygen atoms are located at another plane together with adjacent hydrogen atoms. These planes are tilted to a similar extent to the monoclinic structure, with dihedral angles to the mean plane of each naphthalene group of 39.88 (5) and 40.15 (5)° [mean tilt = 39.83 (5)°]. These numbers differ from those for the monoclinic form by less than 1°.

## Supra­molecular features   

**Figure 5:**
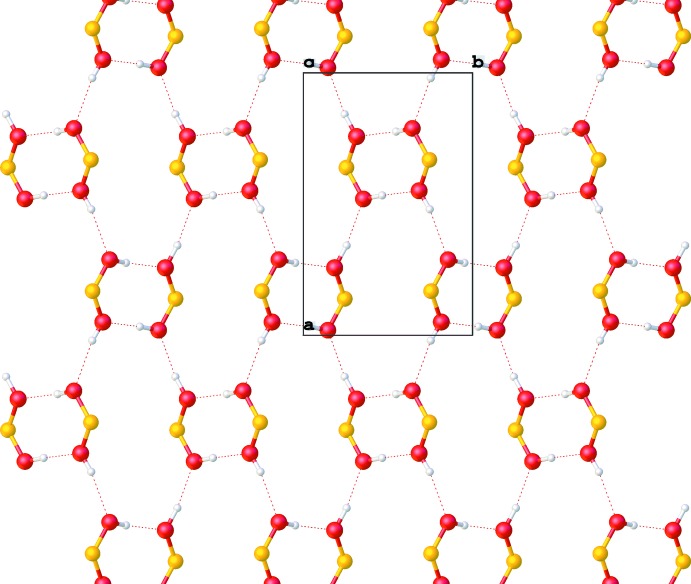
Layered network of hydrogen bonds in the ortho­rhom­bic form. View is along the [001] axis, only boronic acid groups are shown.

**Figure 7:**
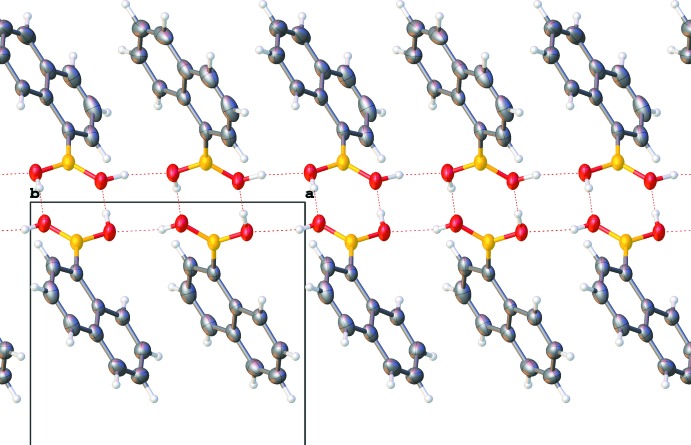
Packing of the ortho­rhom­bic form. View is along the [010] axis.

**Figure 9:**
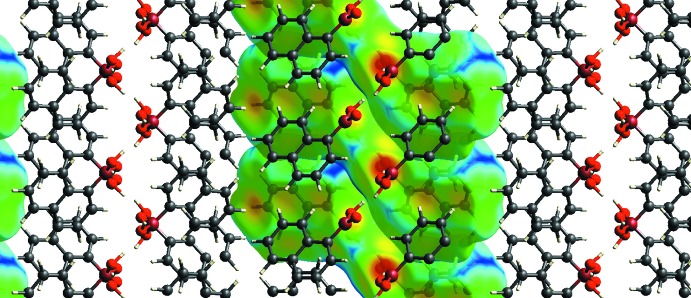
Packing diagram of the ortho­rhom­bic form. View is along the [100] axis. Hirshfeld surface shown for some mol­ecules.

In both forms, pairs of mol­ecules are connected through a pair of O—H⋯O hydrogen bonds (Tables 1 ▸ and 2 ▸) into dimers. There is also an intra­molecular C—H⋯O contact. The dimers are further connected *via* O—H⋯O hydrogen bonds, forming a layered network in plane (001) and in plane (100) in the ortho­rhom­bic and monoclinic forms, respectively (Figs. 5 ▸ and 6 ▸). The resulting layers are practically identical in both forms (compare Figs. 7 ▸ and 8 ▸, Figs. 9 ▸ and 10 ▸).

There are no directional inter­molecular inter­actions between adjacent layers and, therefore, no strong inter­actions between them. However, these layers are shifted with respect to the [010] axis (compare Figs. 9 ▸ and 10 ▸), resulting in a slightly more effective packing of the monoclinic structure (packing index = 0.692) (Kitaigorodskii, 1961 ▸; Spek, 2009 ▸) compared to the ortho­rhom­bic structure (packing index = 0.688). This layer-shift is the only visible difference between the two forms.

## Database survey   

There are no naphthalene boronic acid structures deposited in the Cambridge Structural Database (CSD Version 5.37; Groom *et al.*, 2016 ▸). The simplest aryl­boronic acid, phenyl­boronic acid, crystallizes in a non-centrosymmetric ortho­rhom­bic space group (refcodes PHBORA and PHBORA01). Instead of a layered network, its mol­ecules form an infinitive chain in the crystal (Cyránski *et al.*, 2008 ▸; Rettig & Trotter, 1977 ▸).

## Refinement   

Crystal data, data collection and structure refinement details are summarized in Table 3 ▸. All hydrogen atoms of hydroxyl groups were refined in an isotropic approximation. Aromatic hydrogen atoms were refined with riding coordinates and *U*_iso_(H) = 1.2 *U*_iso_(C).

## Supplementary Material

Crystal structure: contains datablock(s) 1, 2. DOI: 10.1107/S2056989016012494/hb7602sup1.cif

Structure factors: contains datablock(s) 1. DOI: 10.1107/S2056989016012494/hb76021sup2.hkl

Supporting information file. DOI: 10.1107/S2056989016012494/hb76021sup4.cml

Structure factors: contains datablock(s) 2. DOI: 10.1107/S2056989016012494/hb76022sup3.hkl

CCDC references: 1497347, 1497346

Additional supporting information:  crystallographic information; 3D view; checkCIF report

## Figures and Tables

**Figure 3:**
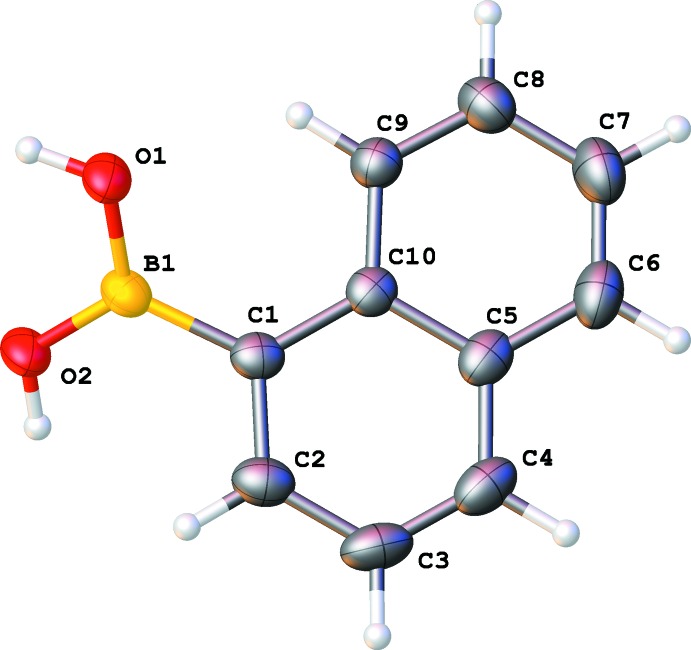
Numbering scheme of the title compound with 50% probability displacement ellipsoids (monoclinic polymorph).

**Figure 6:**
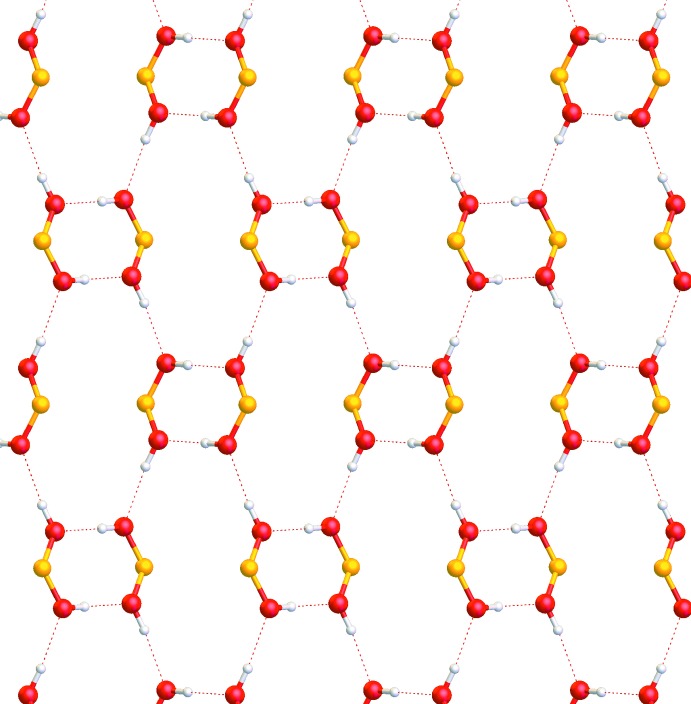
Layered network of hydrogen bonds in the monoclinic form. View is along the [001] axis, only boronic acid groups are shown.

**Figure 8:**
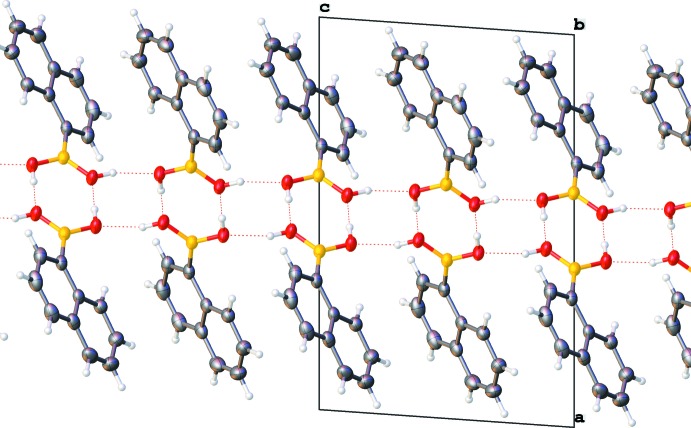
Packing of the monoclinic form. View is along the [010] axis.

**Figure 10:**
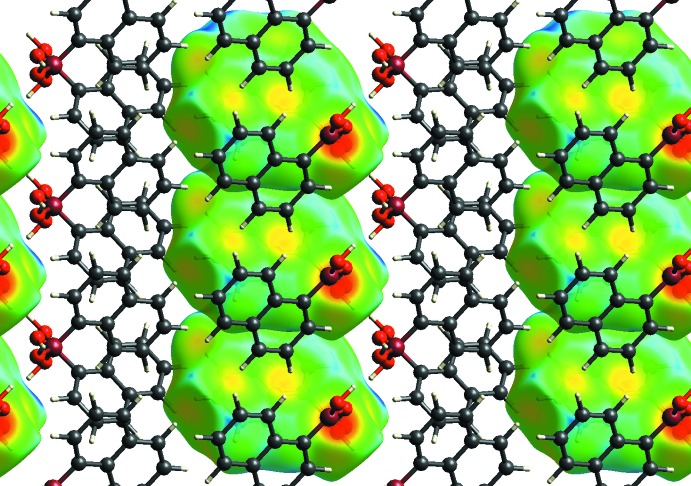
Packing diagram of the monoclinic form. View is along the [001] axis. Hirshfeld surface shown for some mol­ecules.

**Table 1:** Hydrogen-bond geometry (Å, °) for the orthorhombic polymorph[Chem scheme1]

*D*—H⋯*A*	*D*—H	H⋯*A*	*D*⋯*A*	*D*—H⋯*A*
O1—H1⋯O2^i^	0.81 (4)	1.98 (4)	2.766 (2)	165 (4)
O2—H2⋯O3	0.90 (3)	1.86 (3)	2.750 (3)	171 (3)
O3—H3⋯O4^ii^	0.96 (4)	1.82 (4)	2.761 (2)	167 (3)
O4—H4⋯O1	0.89 (4)	1.85 (4)	2.739 (3)	175 (3)
C9—H9⋯O2	0.95	2.45	3.092 (3)	124
C19—H19⋯O4	0.95	2.42	3.063 (3)	125

Symmetry codes: (i) 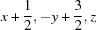; (ii) 
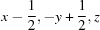
.

**Table 2:** Hydrogen-bond geometry (Å, °) for the monoclinic polymorph[Chem scheme1]

*D*—H⋯*A*	*D*—H	H⋯*A*	*D*⋯*A*	*D*—H⋯*A*
O1—H1⋯O2^i^	0.897 (18)	1.846 (18)	2.7411 (13)	176.3 (17)
O2—H2⋯O1^ii^	0.888 (19)	1.891 (19)	2.7607 (11)	166.0 (17)
C9—H9⋯O1	0.98 (1)	2.43 (1)	3.0911 (15)	124 (1)

Symmetry codes: (i) 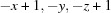; (ii) 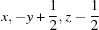.

**Table 3:** Experimental details

	Orthorhombic polymorph	Monoclinic polymorph
Crystal data		
Chemical formula	C_10_H_9_BO_2_	C_10_H_9_BO_2_
*M*_r_	171.98	171.98
Crystal system, space group	Orthorhombic, *P* *n* *a*2_1_	Monoclinic, *P*2_1_/*c*
Temperature (K)	173	173
*a*, *b*, *c* (Å)	9.6655 (4), 6.2286 (3), 29.1778 (13)	14.8469 (11), 6.1023 (4), 9.6797 (7)
α, β, γ (°)	90, 90, 90	90, 93.978 (3), 90
*V* (Å^3^)	1756.58 (14)	874.87 (11)
*Z*	8	4
Radiation type	Cu *K*α	Cu *K*α
μ (mm^−1^)	0.71	0.71
Crystal size (mm)	0.59 × 0.44 × 0.14	0.66 × 0.18 × 0.16

Data collection		
Diffractometer	Bruker PHOTON-100 CMOS	Bruker PHOTON-100 CMOS
Absorption correction	Multi-scan (*SADABS*; Bruker, 2015 ▸)	Multi-scan (*SADABS*; Bruker, 2015)
*T*_min_, *T*_max_	0.671, 0.972	0.759, 0.951
No. of measured, independent and observed [*I* > 2σ(*I*)] reflections	52115, 3764, 3447	25253, 1857, 1576
*R*_int_	0.040	0.038
(sin θ/λ)_max_ (Å^−1^)	0.636	0.633

Refinement		
*R*[*F* ^2^ > 2σ(*F* ^2^)], *wR*(*F* ^2^), *S*	0.035, 0.094, 1.02	0.035, 0.091, 1.04
No. of reflections	3764	1857
No. of parameters	253	133
No. of restraints	1	0
H-atom treatment	H atoms treated by a mixture of independent and constrained refinement	H atoms treated by a mixture of independent and constrained refinement
Δρ_max_, Δρ_min_ (e Å^−3^)	0.22, −0.15	0.23, −0.15
Absolute structure	Flack *x* determined using 1548 quotients [(*I* ^+^)−(*I* ^−^)]/[(*I* ^+^)+(*I* ^−^)] (Parsons et al., 2013 ▸)	–
Absolute structure parameter	0.07 (6)	–

Computer programs: *APEX2* and *SAINT* (Bruker, 2013 ▸), *SHELXT* (Sheldrick, 2015*a* ▸), *SHELXL2014* (Sheldrick, 2015*b* ▸), *OLEX2* (Dolomanov *et al.*, 2009 ▸), *CrystalExplorer* (Spackman & Jayatilaka, 2009 ▸) and *PLATON* (Spek, 2009 ▸).

## supplementary crystallographic information

### (1) (Naphthalen-1-yl)boronic acid Crystal data

**Table:**

C_10_H_9_BO_2_	*D*_x_ = 1.301 Mg m^−^^3^
*M**_r_* = 171.98	Cu *K*α radiation, λ = 1.54178 Å
Orthorhombic, *P**n**a*2_1_	Cell parameters from 9127 reflections
*a* = 9.6655 (4) Å	θ = 3.0–78.2°
*b* = 6.2286 (3) Å	µ = 0.71 mm^−^^1^
*c* = 29.1778 (13) Å	*T* = 173 K
*V* = 1756.58 (14) Å^3^	Plate, colourless
*Z* = 8	0.59 × 0.44 × 0.14 mm
*F*(000) = 720	

### (1) (Naphthalen-1-yl)boronic acid Data collection

**Table:**

Bruker PHOTON-100 CMOS diffractometer	3447 reflections with *I* > 2σ(*I*)
Radiation source: sealedtube	*R*_int_ = 0.040
φ and ω scans	θ_max_ = 78.7°, θ_min_ = 3.0°
Absorption correction: multi-scan (SADABS; Bruker, 2015)	*h* = −12→12
*T*_min_ = 0.671, *T*_max_ = 0.972	*k* = −7→7
52115 measured reflections	*l* = −36→36
3764 independent reflections	

### (1) (Naphthalen-1-yl)boronic acid Refinement

**Table:**

Refinement on *F*^2^	Hydrogen site location: mixed
Least-squares matrix: full	H atoms treated by a mixture of independent and constrained refinement
*R*[*F*^2^ > 2σ(*F*^2^)] = 0.035	*w* = 1/[σ^2^(*F*_o_^2^) + (0.0563*P*)^2^ + 0.2507*P*] where *P* = (*F*_o_^2^ + 2*F*_c_^2^)/3
*wR*(*F*^2^) = 0.094	(Δ/σ)_max_ < 0.001
*S* = 1.02	Δρ_max_ = 0.22 e Å^−^^3^
3764 reflections	Δρ_min_ = −0.15 e Å^−^^3^
253 parameters	Absolute structure: Flack *x* determined using 1548 quotients [(*I*^+^)-(*I*^-^)]/[(*I*^+^)+(*I*^-^)] (Parsons et al., 2013)
1 restraint	Absolute structure parameter: 0.07 (6)

### (1) (Naphthalen-1-yl)boronic acid Special details

**Table:**

Geometry. All esds (except the esd in the dihedral angle between two l.s. planes) are estimated using the full covariance matrix. The cell esds are taken into account individually in the estimation of esds in distances, angles and torsion angles; correlations between esds in cell parameters are only used when they are defined by crystal symmetry. An approximate (isotropic) treatment of cell esds is used for estimating esds involving l.s. planes.

### (1) (Naphthalen-1-yl)boronic acid Fractional atomic coordinates and isotropic or equivalent isotropic displacement parameters (Å^2^)

**Table:**

	*x*	*y*	*z*	*U*_iso_*/*U*_eq_	
O3	0.24283 (16)	0.3150 (3)	0.47433 (6)	0.0415 (4)	
H3	0.158 (4)	0.244 (6)	0.4672 (11)	0.062*	
O4	0.48065 (16)	0.3548 (3)	0.46451 (6)	0.0398 (4)	
H4	0.467 (4)	0.466 (6)	0.4831 (12)	0.060*	
C11	0.3612 (2)	0.0750 (4)	0.41412 (9)	0.0367 (5)	
C12	0.2783 (3)	−0.1033 (4)	0.42012 (11)	0.0472 (6)	
H12	0.222 (2)	−0.1146 (6)	0.4475 (10)	0.057*	
C13	0.2731 (3)	−0.2707 (5)	0.38704 (13)	0.0581 (8)	
H13	0.220 (2)	−0.383 (5)	0.3918 (2)	0.070*	
C14	0.3497 (3)	−0.2587 (5)	0.34829 (12)	0.0549 (8)	
H14	0.3444 (4)	−0.387 (4)	0.3237 (8)	0.066*	
C15	0.4357 (3)	−0.0818 (4)	0.33976 (10)	0.0451 (6)	
C16	0.5152 (3)	−0.0637 (6)	0.29893 (10)	0.0566 (8)	
H16	0.5105 (4)	−0.182 (4)	0.2757 (9)	0.068*	
C17	0.5970 (3)	0.1086 (6)	0.29054 (10)	0.0555 (8)	
H17	0.653 (2)	0.1169 (6)	0.2611 (11)	0.067*	
C18	0.6038 (3)	0.2752 (5)	0.32268 (9)	0.0485 (7)	
H18	0.666 (2)	0.407 (5)	0.3163 (2)	0.058*	
C19	0.5287 (2)	0.2649 (4)	0.36254 (8)	0.0389 (5)	
H19	0.5350	0.3799	0.3838	0.047*	
C20	0.4422 (2)	0.0882 (4)	0.37291 (8)	0.0358 (5)	
B2	0.3611 (3)	0.2548 (5)	0.45182 (10)	0.0352 (6)	
O1	0.44946 (17)	0.6845 (3)	0.52575 (6)	0.0410 (4)	
H1	0.520 (4)	0.747 (6)	0.5329 (13)	0.061*	
O2	0.21178 (16)	0.6447 (3)	0.53618 (6)	0.0393 (4)	
H2	0.224 (4)	0.548 (5)	0.5138 (12)	0.059*	
C1	0.3326 (2)	0.9282 (4)	0.58539 (8)	0.0337 (5)	
C2	0.4169 (2)	1.1032 (4)	0.57782 (10)	0.0417 (6)	
H2A	0.4712	1.1076	0.5507	0.050*	
C3	0.4253 (3)	1.2758 (4)	0.60894 (11)	0.0484 (7)	
H3A	0.4842	1.3941	0.6025	0.058*	
C4	0.3491 (3)	1.2734 (4)	0.64824 (11)	0.0460 (6)	
H4A	0.3552	1.3901	0.6691	0.055*	
C5	0.2613 (2)	1.0981 (4)	0.65810 (9)	0.0386 (5)	
C6	0.1826 (3)	1.0920 (5)	0.69941 (9)	0.0462 (6)	
H6	0.1876	1.2092	0.7202	0.055*	
C7	0.1010 (3)	0.9220 (5)	0.70950 (9)	0.0482 (6)	
H7	0.0499	0.9201	0.7373	0.058*	
C8	0.0916 (3)	0.7484 (5)	0.67893 (9)	0.0437 (6)	
H8	0.0343	0.6294	0.6862	0.052*	
C9	0.1647 (2)	0.7492 (4)	0.63858 (8)	0.0361 (5)	
H9	0.1567	0.6308	0.6182	0.043*	
C10	0.2520 (2)	0.9235 (4)	0.62674 (8)	0.0328 (5)	
B1	0.3317 (3)	0.7451 (5)	0.54832 (9)	0.0347 (5)	

### (1) (Naphthalen-1-yl)boronic acid Atomic displacement parameters (Å^2^)

**Table:**

	*U*^11^	*U*^22^	*U*^33^	*U*^12^	*U*^13^	*U*^23^
O3	0.0220 (7)	0.0608 (11)	0.0418 (9)	−0.0033 (8)	0.0009 (6)	−0.0085 (8)
O4	0.0227 (7)	0.0537 (10)	0.0430 (10)	−0.0004 (8)	−0.0010 (6)	−0.0104 (8)
C11	0.0239 (10)	0.0383 (12)	0.0479 (13)	0.0028 (9)	−0.0073 (9)	−0.0001 (11)
C12	0.0329 (11)	0.0453 (14)	0.0633 (17)	−0.0010 (11)	−0.0080 (12)	0.0086 (12)
C13	0.0457 (15)	0.0360 (13)	0.093 (3)	−0.0063 (12)	−0.0258 (16)	0.0035 (14)
C14	0.0487 (16)	0.0426 (14)	0.073 (2)	0.0077 (12)	−0.0200 (14)	−0.0160 (14)
C15	0.0370 (12)	0.0442 (14)	0.0541 (16)	0.0115 (10)	−0.0158 (11)	−0.0128 (12)
C16	0.0511 (16)	0.0719 (19)	0.0469 (16)	0.0244 (15)	−0.0136 (12)	−0.0248 (15)
C17	0.0450 (15)	0.079 (2)	0.0421 (15)	0.0145 (15)	−0.0012 (11)	−0.0093 (14)
C18	0.0381 (14)	0.0660 (18)	0.0414 (14)	0.0042 (13)	−0.0002 (10)	−0.0029 (12)
C19	0.0309 (12)	0.0461 (13)	0.0396 (12)	0.0038 (10)	−0.0037 (10)	−0.0061 (10)
C20	0.0270 (9)	0.0387 (12)	0.0417 (13)	0.0079 (9)	−0.0074 (9)	−0.0064 (10)
B2	0.0231 (12)	0.0454 (14)	0.0371 (13)	0.0006 (11)	−0.0011 (10)	0.0038 (11)
O1	0.0234 (7)	0.0568 (11)	0.0427 (10)	−0.0046 (8)	0.0025 (6)	−0.0069 (8)
O2	0.0212 (7)	0.0542 (10)	0.0424 (9)	0.0002 (7)	0.0011 (6)	−0.0093 (8)
C1	0.0242 (9)	0.0371 (11)	0.0399 (12)	0.0030 (9)	−0.0045 (8)	0.0034 (10)
C2	0.0284 (11)	0.0433 (13)	0.0533 (15)	−0.0009 (10)	−0.0037 (10)	0.0086 (11)
C3	0.0369 (14)	0.0342 (12)	0.0741 (19)	−0.0046 (11)	−0.0109 (13)	0.0058 (12)
C4	0.0382 (14)	0.0351 (12)	0.0647 (18)	0.0031 (11)	−0.0148 (11)	−0.0056 (12)
C5	0.0313 (11)	0.0391 (12)	0.0453 (14)	0.0080 (10)	−0.0107 (9)	−0.0030 (10)
C6	0.0454 (14)	0.0501 (14)	0.0430 (14)	0.0113 (12)	−0.0084 (11)	−0.0114 (11)
C7	0.0440 (14)	0.0630 (16)	0.0377 (13)	0.0078 (13)	0.0009 (10)	−0.0025 (12)
C8	0.0379 (13)	0.0500 (14)	0.0433 (13)	0.0023 (12)	0.0010 (10)	0.0045 (10)
C9	0.0303 (11)	0.0386 (11)	0.0395 (12)	0.0016 (9)	−0.0025 (9)	−0.0011 (9)
C10	0.0248 (9)	0.0345 (11)	0.0392 (12)	0.0050 (9)	−0.0070 (8)	0.0009 (9)
B1	0.0228 (12)	0.0439 (13)	0.0374 (13)	0.0014 (10)	−0.0004 (9)	0.0007 (11)

### (1) (Naphthalen-1-yl)boronic acid Geometric parameters (Å, º)

**Table:**

O3—H3	0.95 (4)	O1—H1	0.81 (4)
O3—B2	1.371 (3)	O1—B1	1.368 (3)
O4—H4	0.89 (4)	O2—H2	0.90 (3)
O4—B2	1.363 (3)	O2—B1	1.364 (3)
C11—C12	1.381 (4)	C1—C2	1.379 (4)
C11—C20	1.438 (3)	C1—C10	1.436 (3)
C11—B2	1.570 (4)	C1—B1	1.572 (4)
C12—H12	0.97 (3)	C2—H2A	0.9500
C12—C13	1.422 (5)	C2—C3	1.410 (4)
C13—H13	0.88 (4)	C3—H3A	0.9500
C13—C14	1.353 (5)	C3—C4	1.363 (4)
C14—H14	1.07 (4)	C4—H4A	0.9500
C14—C15	1.403 (4)	C4—C5	1.413 (4)
C15—C16	1.422 (4)	C5—C6	1.426 (4)
C15—C20	1.435 (3)	C5—C10	1.424 (3)
C16—H16	1.00 (4)	C6—H6	0.9500
C16—C17	1.355 (5)	C6—C7	1.353 (4)
C17—H17	1.02 (4)	C7—H7	0.9500
C17—C18	1.400 (4)	C7—C8	1.405 (4)
C18—H18	1.03 (4)	C8—H8	0.9500
C18—C19	1.373 (3)	C8—C9	1.373 (4)
C19—H19	0.9500	C9—H9	0.9500
C19—C20	1.415 (3)	C9—C10	1.417 (3)
			
B2—O3—H3	119 (2)	B1—O1—H1	116 (3)
B2—O4—H4	113 (2)	B1—O2—H2	113 (2)
C12—C11—C20	117.9 (2)	C2—C1—C10	118.1 (2)
C12—C11—B2	119.0 (2)	C2—C1—B1	117.8 (2)
C20—C11—B2	123.1 (2)	C10—C1—B1	124.1 (2)
C11—C12—H12	119.2	C1—C2—H2A	118.9
C11—C12—C13	121.6 (3)	C1—C2—C3	122.3 (3)
C13—C12—H12	119.2	C3—C2—H2A	118.9
C12—C13—H13	119.7	C2—C3—H3A	119.9
C14—C13—C12	120.5 (3)	C4—C3—C2	120.1 (2)
C14—C13—H13	119.7	C4—C3—H3A	119.9
C13—C14—H14	119.5	C3—C4—H4A	119.9
C13—C14—C15	121.0 (3)	C3—C4—C5	120.3 (3)
C15—C14—H14	119.5	C5—C4—H4A	119.9
C14—C15—C16	122.1 (3)	C4—C5—C6	120.9 (2)
C14—C15—C20	119.1 (3)	C4—C5—C10	119.8 (2)
C16—C15—C20	118.8 (3)	C10—C5—C6	119.3 (2)
C15—C16—H16	119.0	C5—C6—H6	119.5
C17—C16—C15	122.0 (3)	C7—C6—C5	121.1 (2)
C17—C16—H16	119.0	C7—C6—H6	119.5
C16—C17—H17	120.2	C6—C7—H7	119.9
C16—C17—C18	119.6 (3)	C6—C7—C8	120.1 (3)
C18—C17—H17	120.2	C8—C7—H7	119.9
C17—C18—H18	119.7	C7—C8—H8	119.7
C19—C18—C17	120.5 (3)	C9—C8—C7	120.6 (3)
C19—C18—H18	119.7	C9—C8—H8	119.7
C18—C19—H19	119.0	C8—C9—H9	119.4
C18—C19—C20	122.0 (2)	C8—C9—C10	121.2 (2)
C20—C19—H19	119.0	C10—C9—H9	119.4
C15—C20—C11	119.8 (2)	C5—C10—C1	119.3 (2)
C19—C20—C11	123.0 (2)	C9—C10—C1	122.9 (2)
C19—C20—C15	117.1 (2)	C9—C10—C5	117.8 (2)
O3—B2—C11	122.1 (2)	O1—B1—C1	121.8 (2)
O4—B2—O3	116.9 (2)	O2—B1—O1	117.1 (2)
O4—B2—C11	121.1 (2)	O2—B1—C1	121.0 (2)
			
C11—C12—C13—C14	−0.4 (4)	C1—C2—C3—C4	0.3 (4)
C12—C11—C20—C15	−0.3 (3)	C2—C1—C10—C5	0.2 (3)
C12—C11—C20—C19	178.6 (2)	C2—C1—C10—C9	−178.3 (2)
C12—C11—B2—O3	−38.2 (3)	C2—C1—B1—O1	37.5 (3)
C12—C11—B2—O4	140.1 (2)	C2—C1—B1—O2	−140.3 (2)
C12—C13—C14—C15	0.0 (4)	C2—C3—C4—C5	0.0 (4)
C13—C14—C15—C16	−178.8 (3)	C3—C4—C5—C6	179.0 (2)
C13—C14—C15—C20	0.2 (4)	C3—C4—C5—C10	−0.2 (3)
C14—C15—C16—C17	179.2 (3)	C4—C5—C6—C7	−178.4 (2)
C14—C15—C20—C11	0.0 (3)	C4—C5—C10—C1	0.1 (3)
C14—C15—C20—C19	−179.1 (2)	C4—C5—C10—C9	178.7 (2)
C15—C16—C17—C18	−0.3 (4)	C5—C6—C7—C8	−0.5 (4)
C16—C15—C20—C11	179.0 (2)	C6—C5—C10—C1	−179.2 (2)
C16—C15—C20—C19	0.0 (3)	C6—C5—C10—C9	−0.6 (3)
C16—C17—C18—C19	0.2 (4)	C6—C7—C8—C9	−0.1 (4)
C17—C18—C19—C20	0.0 (4)	C7—C8—C9—C10	0.4 (4)
C18—C19—C20—C11	−179.1 (2)	C8—C9—C10—C1	178.5 (2)
C18—C19—C20—C15	−0.1 (3)	C8—C9—C10—C5	0.0 (3)
C20—C11—C12—C13	0.5 (3)	C10—C1—C2—C3	−0.4 (3)
C20—C11—B2—O3	140.8 (2)	C10—C1—B1—O1	−142.0 (2)
C20—C11—B2—O4	−41.0 (3)	C10—C1—B1—O2	40.3 (3)
C20—C15—C16—C17	0.2 (4)	C10—C5—C6—C7	0.9 (3)
B2—C11—C12—C13	179.6 (2)	B1—C1—C2—C3	−179.9 (2)
B2—C11—C20—C15	−179.3 (2)	B1—C1—C10—C5	179.7 (2)
B2—C11—C20—C19	−0.3 (3)	B1—C1—C10—C9	1.2 (3)

### (1) (Naphthalen-1-yl)boronic acid Hydrogen-bond geometry (Å, º)

**Table:**

*D*—H···*A*	*D*—H	H···*A*	*D*···*A*	*D*—H···*A*
O1—H1···O2^i^	0.81 (4)	1.98 (4)	2.766 (2)	165 (4)
O2—H2···O3	0.90 (3)	1.86 (3)	2.750 (3)	171 (3)
O3—H3···O4^ii^	0.96 (4)	1.82 (4)	2.761 (2)	167 (3)
O4—H4···O1	0.89 (4)	1.85 (4)	2.739 (3)	175 (3)
C9—H9···O2	0.95	2.45	3.092 (3)	124
C19—H19···O4	0.95	2.42	3.063 (3)	125

Symmetry codes: (i) *x*+1/2, −*y*+3/2, *z*; (ii) *x*−1/2, −*y*+1/2, *z*.

### (2) (Naphthalen-1-yl)boronic acid Crystal data

**Table:**

C_10_H_9_BO_2_	*F*(000) = 360
*M**_r_* = 171.98	*D*_x_ = 1.306 Mg m^−^^3^
Monoclinic, *P*2_1_/*c*	Cu *K*α radiation, λ = 1.54178 Å
*a* = 14.8469 (11) Å	Cell parameters from 9898 reflections
*b* = 6.1023 (4) Å	θ = 3.0–77.0°
*c* = 9.6797 (7) Å	µ = 0.71 mm^−^^1^
β = 93.978 (3)°	*T* = 173 K
*V* = 874.87 (11) Å^3^	Prism, colourless
*Z* = 4	0.66 × 0.18 × 0.16 mm

### (2) (Naphthalen-1-yl)boronic acid Data collection

**Table:**

Bruker PHOTON-100 CMOS diffractometer	1576 reflections with *I* > 2σ(*I*)
Radiation source: sealedtube	*R*_int_ = 0.038
φ and ω scans	θ_max_ = 77.4°, θ_min_ = 3.0°
Absorption correction: multi-scan (SADABS; Bruker, 2015)	*h* = −18→18
*T*_min_ = 0.759, *T*_max_ = 0.951	*k* = −7→7
25253 measured reflections	*l* = −12→11
1857 independent reflections	

### (2) (Naphthalen-1-yl)boronic acid Refinement

**Table:**

Refinement on *F*^2^	0 restraints
Least-squares matrix: full	Hydrogen site location: mixed
*R*[*F*^2^ > 2σ(*F*^2^)] = 0.035	H atoms treated by a mixture of independent and constrained refinement
*wR*(*F*^2^) = 0.091	*w* = 1/[σ^2^(*F*_o_^2^) + (0.0435*P*)^2^ + 0.2015*P*] where *P* = (*F*_o_^2^ + 2*F*_c_^2^)/3
*S* = 1.04	(Δ/σ)_max_ < 0.001
1857 reflections	Δρ_max_ = 0.23 e Å^−^^3^
133 parameters	Δρ_min_ = −0.15 e Å^−^^3^

### (2) (Naphthalen-1-yl)boronic acid Special details

**Table:**

Geometry. All esds (except the esd in the dihedral angle between two l.s. planes) are estimated using the full covariance matrix. The cell esds are taken into account individually in the estimation of esds in distances, angles and torsion angles; correlations between esds in cell parameters are only used when they are defined by crystal symmetry. An approximate (isotropic) treatment of cell esds is used for estimating esds involving l.s. planes.

### (2) (Naphthalen-1-yl)boronic acid Fractional atomic coordinates and isotropic or equivalent isotropic displacement parameters (Å^2^)

**Table:**

	*x*	*y*	*z*	*U*_iso_*/*U*_eq_	
O1	0.42757 (6)	0.14734 (15)	0.62491 (7)	0.0348 (2)	
H1	0.4697 (12)	0.043 (3)	0.6194 (17)	0.058 (5)*	
O2	0.44930 (6)	0.18505 (15)	0.39004 (8)	0.0361 (2)	
H2	0.4335 (12)	0.247 (3)	0.309 (2)	0.067 (5)*	
C1	0.32826 (8)	0.42799 (19)	0.49000 (11)	0.0301 (3)	
C2	0.34171 (9)	0.6083 (2)	0.40723 (12)	0.0374 (3)	
H2A	0.3975 (9)	0.6190 (3)	0.3608 (7)	0.045*	
C3	0.27729 (10)	0.7770 (2)	0.38806 (13)	0.0441 (3)	
H3	0.2893 (2)	0.903 (2)	0.3287 (10)	0.053*	
C4	0.19841 (10)	0.7660 (2)	0.45170 (13)	0.0431 (3)	
H4	0.1527 (8)	0.887 (2)	0.4375 (3)	0.052*	
C5	0.18023 (8)	0.5861 (2)	0.53761 (12)	0.0356 (3)	
C6	0.09731 (9)	0.5709 (2)	0.60194 (14)	0.0445 (3)	
H6	0.0525 (8)	0.688 (2)	0.5877 (3)	0.053*	
C7	0.07934 (9)	0.3962 (3)	0.68300 (14)	0.0477 (3)	
H7	0.0219 (10)	0.3879 (3)	0.7264 (8)	0.057*	
C8	0.14332 (9)	0.2281 (2)	0.70431 (13)	0.0411 (3)	
H8	0.1299 (2)	0.100 (2)	0.7651 (10)	0.049*	
C9	0.22382 (8)	0.2367 (2)	0.64330 (11)	0.0327 (3)	
H9	0.2672 (6)	0.1175 (18)	0.6589 (3)	0.039*	
C10	0.24527 (8)	0.41549 (18)	0.55791 (11)	0.0296 (3)	
B1	0.40328 (9)	0.2462 (2)	0.50178 (12)	0.0300 (3)	

### (2) (Naphthalen-1-yl)boronic acid Atomic displacement parameters (Å^2^)

**Table:**

	*U*^11^	*U*^22^	*U*^33^	*U*^12^	*U*^13^	*U*^23^
O1	0.0376 (5)	0.0461 (5)	0.0209 (4)	0.0093 (4)	0.0038 (3)	0.0008 (3)
O2	0.0375 (5)	0.0500 (5)	0.0212 (4)	0.0078 (4)	0.0055 (3)	0.0041 (4)
C1	0.0372 (6)	0.0315 (6)	0.0212 (5)	−0.0021 (5)	−0.0008 (4)	−0.0028 (4)
C2	0.0476 (7)	0.0370 (6)	0.0274 (6)	−0.0065 (5)	0.0009 (5)	0.0002 (5)
C3	0.0669 (9)	0.0306 (6)	0.0333 (6)	−0.0033 (6)	−0.0060 (6)	0.0046 (5)
C4	0.0575 (8)	0.0333 (6)	0.0367 (7)	0.0098 (6)	−0.0093 (6)	−0.0037 (5)
C5	0.0429 (7)	0.0357 (6)	0.0271 (6)	0.0062 (5)	−0.0058 (5)	−0.0069 (5)
C6	0.0384 (7)	0.0532 (8)	0.0413 (7)	0.0139 (6)	−0.0034 (5)	−0.0098 (6)
C7	0.0334 (7)	0.0664 (9)	0.0434 (7)	0.0036 (6)	0.0047 (5)	−0.0042 (7)
C8	0.0368 (6)	0.0500 (7)	0.0366 (7)	−0.0038 (6)	0.0036 (5)	0.0016 (6)
C9	0.0335 (6)	0.0348 (6)	0.0293 (6)	0.0010 (5)	−0.0007 (4)	−0.0010 (5)
C10	0.0349 (6)	0.0311 (6)	0.0221 (5)	0.0009 (5)	−0.0022 (4)	−0.0051 (4)
B1	0.0308 (6)	0.0366 (7)	0.0226 (6)	−0.0031 (5)	0.0024 (4)	−0.0013 (5)

### (2) (Naphthalen-1-yl)boronic acid Geometric parameters (Å, º)

**Table:**

O1—H1	0.899 (19)	C4—C5	1.4146 (18)
O1—B1	1.3620 (15)	C5—C6	1.4206 (19)
O2—H2	0.886 (19)	C5—C10	1.4243 (16)
O2—B1	1.3706 (14)	C6—H6	0.979 (16)
C1—C2	1.3839 (16)	C6—C7	1.361 (2)
C1—C10	1.4382 (16)	C7—H7	0.977 (16)
C1—B1	1.5705 (18)	C7—C8	1.4036 (19)
C2—H2A	0.971 (15)	C8—H8	1.008 (16)
C2—C3	1.4085 (19)	C8—C9	1.3703 (17)
C3—H3	0.983 (16)	C9—H9	0.977 (14)
C3—C4	1.362 (2)	C9—C10	1.4180 (16)
C4—H4	1.004 (16)		
			
B1—O1—H1	114.0 (10)	C7—C6—C5	120.98 (12)
B1—O2—H2	117.8 (11)	C7—C6—H6	119.5
C2—C1—C10	118.00 (11)	C6—C7—H7	120.0
C2—C1—B1	118.27 (11)	C6—C7—C8	119.95 (12)
C10—C1—B1	123.72 (10)	C8—C7—H7	120.0
C1—C2—H2A	118.9	C7—C8—H8	119.7
C1—C2—C3	122.23 (12)	C9—C8—C7	120.68 (13)
C3—C2—H2A	118.9	C9—C8—H8	119.7
C2—C3—H3	119.9	C8—C9—H9	119.4
C4—C3—C2	120.12 (12)	C8—C9—C10	121.23 (11)
C4—C3—H3	119.9	C10—C9—H9	119.4
C3—C4—H4	119.7	C5—C10—C1	119.49 (11)
C3—C4—C5	120.60 (12)	C9—C10—C1	122.78 (10)
C5—C4—H4	119.7	C9—C10—C5	117.72 (11)
C4—C5—C6	121.00 (12)	O1—B1—O2	116.98 (11)
C4—C5—C10	119.56 (12)	O1—B1—C1	121.32 (10)
C6—C5—C10	119.43 (12)	O2—B1—C1	121.66 (10)
C5—C6—H6	119.5		
			
C1—C2—C3—C4	−0.16 (19)	C6—C5—C10—C9	−0.34 (16)
C2—C1—C10—C5	0.31 (15)	C6—C7—C8—C9	−0.5 (2)
C2—C1—C10—C9	179.01 (10)	C7—C8—C9—C10	0.45 (18)
C2—C1—B1—O1	139.56 (12)	C8—C9—C10—C1	−178.73 (11)
C2—C1—B1—O2	−38.24 (16)	C8—C9—C10—C5	−0.01 (16)
C2—C3—C4—C5	0.06 (19)	C10—C1—C2—C3	−0.04 (17)
C3—C4—C5—C6	−178.59 (12)	C10—C1—B1—O1	−41.91 (16)
C3—C4—C5—C10	0.22 (18)	C10—C1—B1—O2	140.30 (11)
C4—C5—C6—C7	179.08 (12)	C10—C5—C6—C7	0.27 (18)
C4—C5—C10—C1	−0.41 (16)	B1—C1—C2—C3	178.58 (11)
C4—C5—C10—C9	−179.17 (10)	B1—C1—C10—C5	−178.22 (10)
C5—C6—C7—C8	0.2 (2)	B1—C1—C10—C9	0.47 (16)
C6—C5—C10—C1	178.42 (10)		

### (2) (Naphthalen-1-yl)boronic acid Hydrogen-bond geometry (Å, º)

**Table:**

*D*—H···*A*	*D*—H	H···*A*	*D*···*A*	*D*—H···*A*
O1—H1···O2^i^	0.897 (18)	1.846 (18)	2.7411 (13)	176.3 (17)
O2—H2···O1^ii^	0.888 (19)	1.891 (19)	2.7607 (11)	166.0 (17)
C9—H9···O1	0.98 (1)	2.43 (1)	3.0911 (15)	124 (1)

Symmetry codes: (i) −*x*+1, −*y*, −*z*+1; (ii) *x*, −*y*+1/2, *z*−1/2.
